# ‘I want to feel at home’: establishing what aspects of environmental design are important to people with dementia nearing the end of life

**DOI:** 10.1186/s12904-015-0026-y

**Published:** 2015-05-12

**Authors:** Richard Fleming, Fiona Kelly, Gillian Stillfried

**Affiliations:** School of Nursing, University of Wollongong, Northfields Avenue, Wollongong, Australia; Bournemouth University Dementia Institute, Bournemouth, UK

## Abstract

**Background:**

The design of environments in which people with dementia live should be understandable, reinforce personal identity and maintain their abilities. The focus on supporting people with dementia to live well has omitted considering the needs or wishes for a supportive physical environment of those who are nearing the end of their lives. Using a combination of focus groups and a Delphi survey, this study explored the views of people with dementia, family carers and professionals on what aspects of the physical environment would be important to support a good quality of life to the very end.

**Methods:**

Three focus groups were carried out in three cities along the East Coast of Australia using a discussion guide informed by a literature review. Focus groups comprised recently bereaved family carers of people with dementia (FG1), people with dementia and family carers of people with dementia (FG2) and practitioners caring for people with dementia nearing or at the end of their lives (FG3). Focus group conversations were audio-recorded with participants’ consent. Audio files were transcribed verbatim and analysed thematically to identify environmental features that could contribute to achieving the goal of providing a comfortable life to the end. A list of design features derived from analysis of focus group transcripts was distributed to a range of experts in the dementia field and a consensus sought on their appropriateness. From this, a set of features to inform the design of environments for people with dementia nearing the end of life was defined.

**Results:**

Eighteen people took part in three focus groups: two with dementia, eleven current or recently bereaved family carers and five practitioners. There were differences in opinion on what were important environmental considerations. People with dementia and family carers identified comfort through engagement, feeling at home, a calm environment, privacy and dignity and use of technology to remain connected as important. For practitioners, design to facilitate duty of care and institutional influences on their practice were salient themes. Twenty one experts in the dementia field took part in the survey to agree a consensus on the desirable features derived from analysis of focus group transcripts, with fifteen features agreed.

**Conclusions:**

The fifteen features are compatible with the design principles for people with dementia who are mobile, but include a stronger focus on sensory engagement. We suggest that considering these features as part of a continuum of care will support good practice and offer those with dementia the opportunity to live well until the end and give their families a more positive experience in the last days of their lives together.

## Background

There are growing calls for end of life care to be provided, not just to those with cancer [1, 2] but for people with coronary heart disease, older people [3] and indeed all people approaching the end of life regardless of age, diagnosis, gender, ethnicity, sexual orientation, religious belief, disability or socio-economic status [4]. In the UK, the Department of Health [4] also proposes that high quality care at end of life should be available wherever the person may be – at home, in a care home, a hospice, hospital or elsewhere.

The case for making palliative care available for people with dementia has been made on the grounds of equity, need and on the basis that adopting a palliative approach would improve the quality of care available to people with dementia; throughout their journey [5]. In the UK, the philosophy of palliative care emphasizes care and communication over inappropriate intervention and treatment [6]; it attempts to redirect the emphasis on technology-driven medicine. The European Association for Palliative Care, in their white paper, reached a consensus on eleven domains of optimal palliative care for people with dementia, including ‘for dying people, a comfortable environment is desirable’ [7], however there is no elaboration on what this might entail. In Australia, guidance on palliative and end of life care emphasizes a person-centered [8] approach which meets physical, psycho-social and spiritual needs and addresses aspects of the environment such as ensuring bedrooms are of sufficient size to accommodate visitors and equipment, and that sensory support is offered [9].

The association between advanced age and dementia indicates a rapidly increasing prevalence of people with dementia resident in the care home sector. In Australia during 2006-2007, 72.7 % of people admitted to care homes were 80+ years of age, an increase from 64.1 % in 1998-1999 [10]. Worldwide it is estimated that four fifths of people in care homes have a dementia [11]. The National End of Life Care Intelligence Network report that the largest percentages of deaths of people with dementia occur in hospital (36%), followed by nursing homes (30%) and supported accommodation (26%) [12]. Relatively small percentages die in their own homes, in hospices, or elsewhere.

The increasing numbers of people entering care homes in a frailer state and policy drivers to provide high quality palliative and end of life care to people with dementia [4, 13] have prompted consideration of what a palliative approach for dementia care entails [7]. This paper explores one aspect of care for people with dementia nearing the end of life: what environmental characteristics are important to promote living as well as possible until death [14].

A ‘dementia friendly’ environment has been described as ‘a cohesive system of support that recognises the experiences of the person with dementia and best provides assistance for the person to remain engaged in everyday life in a meaningful way’ (p.187) [15]. Any definition of a dementia friendly environment should consider both the experiences of the person with dementia within the environment and also the social, physical and organisational environments which impact on these experiences. Lyman (p.15) [16] states that ‘care providers and care recipients inhabit the unique world of dementia care. If designers and programme planners can understand this world from the perspective of persons living with dementia, an “enabling” environment can minimise disability and provide opportunities to live a meaningful life, despite losses and challenges associated [with dementia].’

The importance of ensuring that the design of buildings meets the needs of people with dementia and makes sense to them has been championed by Marshall [17]. Marshall’s summary of the key principles of design and dementia continues to be used as the quality standard of good design, with substantial empirical support for these principles generated in subsequent years [18]. She asserted that design should:

- Compensate for disability
- Maximise independence
- Enhance self-esteem and confidence
- Demonstrate care for staff
- Be orientating and understandable
- Reinforce personal identity
- Welcome relatives and the local community
- Allow for the control of stimuli.

A further set of principles for the design of care settings for older people with dementia have been developed [19, 20]. These principles state that environments that are used to provide care ‘aimed at maintaining the abilities of people with dementia should’:

- Be safe and secure
- Be small
- Be simple and provide good ‘visual access’
- Reduce unwanted stimulation
- Highlight helpful stimuli
- Provide for planned wandering
- Be familiar
- Provide a variety of spaces with opportunities for both privacy and community
- Provide links to the community
- Be domestic and homelike.

Such principles allow staff and management to gain an understanding of problems that are caused by the environment in which people with dementia live, allowing for the implementation of short and long-term plans for improving the environment to support care [21].

The focus of “dementia friendly environments” [15] has been on maintaining independence and well-being through engagement and is therefore aimed at those who are relatively fit and mobile. Despite the relatively large body of work on identifying optimum design principles for people with dementia [22, 18], with a focus on maintaining independence and balancing sensory stimulation according to needs, there is little literature and even less research into design that focuses on the needs of those with advanced dementia [23], or those with dementia who are nearing the end of life or dying. With an increasingly frail population of people with dementia receiving care in formal care settings, current design principles might not accurately reflect this population’s needs and wishes; this might particularly be the case for people with dementia approaching the end of their lives. The overall aim of this study was to identify the environmental features that are desirable in buildings used to provide care for people with dementia nearing the end of their lives.

The specific objectives of this research were to:

- gain a better understanding of the needs and wishes of people with dementia nearing the end of their lives, and those of their families,
- gain a better understanding of the physical resources required by the staff caring for them and
- identify a set of features that will inform the design of physical environments that accommodate the needs of people with dementia nearing the end of their lives.

**Fig 1:**
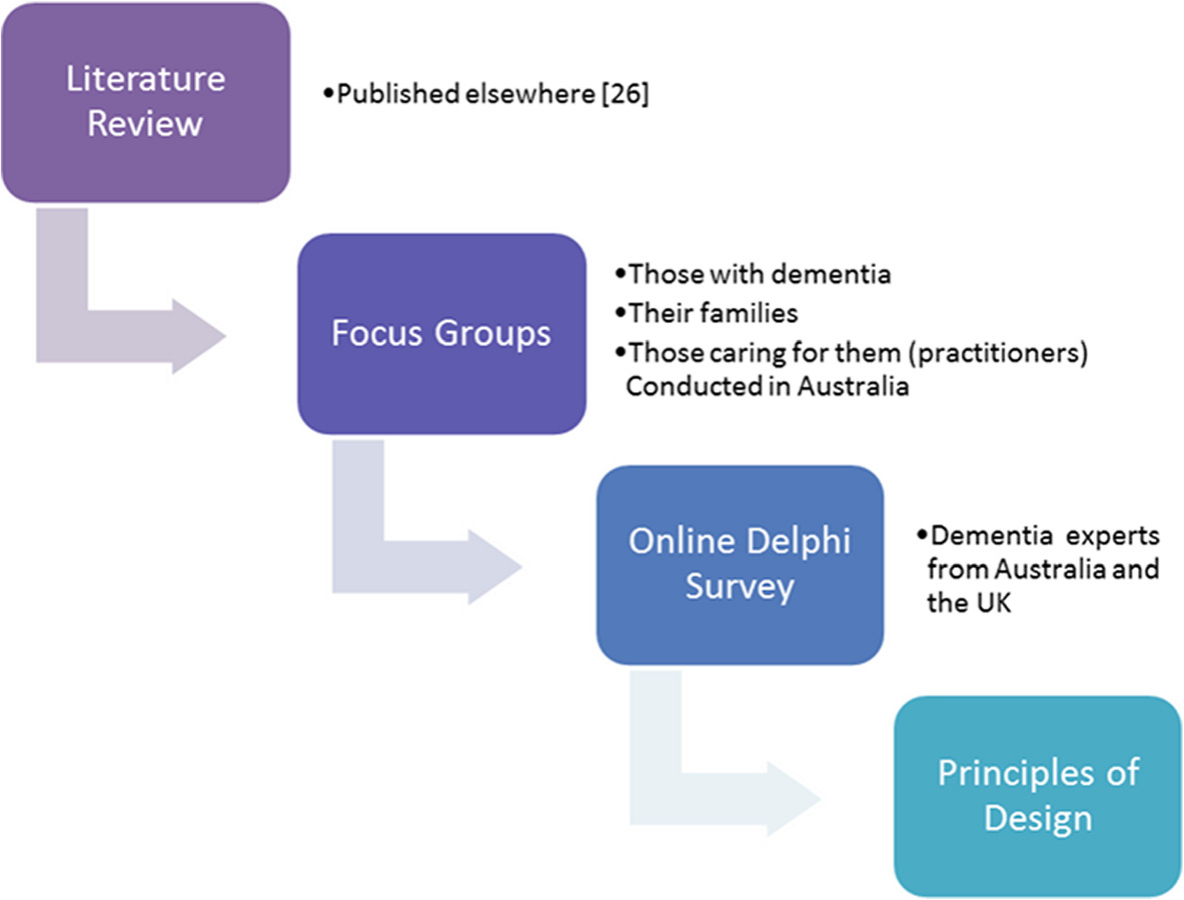
Mixed methods approach used in this study

We aimed to reach these objectives by using a mixed methods design (Fig. 1). Ethical approval for the study was obtained from the University of Wollongong/South Eastern Sydney and Illawarra Area Health Service Human Research Ethics Committee (Australia) (Protocol number HE11/265). Ethical processes were followed to ensure informed consent, anonymity, confidentiality and prevention of harm.

## Methods

The end of the person with dementia’s life was conceptualised as occurring in the severe stage of dementia [24]. This stage is characterised by severe memory loss, disorientation to time and place, inability to function independently outside of the home, very restricted or no interests and requiring total assistance with personal care.

### Focus groups

Focus groups are a useful method to identify a range of opinions by discussing a particular issue with a small group of people [25]. Focus groups were used to explore the needs and wishes of those with dementia, their families and those caring for them (practitioners) with respect to design of environments when nearing the end of life. The findings from a literature review [26] were used to generate a list of topics to guide the focus group discussions (Fig. 1).

Three focus groups (FG) were carried out by two of the research team RF and FK in three cities along the East coast of Australia during 2012. Both RF and FK are experienced researchers who have conducted numerous participant interviews and focus groups [27-31] and have established research backgrounds in environmental design for people living with dementia [19-21, 18, 27, 28].

A convenience sample of participants with dementia and family carers were recruited via the Alzheimer’s Australia Consumer Dementia Research Network. Members of the Dementia Research Network are people with dementia who volunteer to provide comments on research applications and to offer suggestions for areas of research. Practitioners were recruited by invitation via the New South Wales and Australian Capital Territory Dementia Training Study Centre mailing list. FG1 involved four recently bereaved family carers of people with dementia: all female. Three were the wives of recently deceased people with dementia, the fourth was the daughter of a person with dementia in residential aged care. FG2 involved nine participants: seven female and two male. Five were the spouses of deceased people with dementia, two were daughters of deceased people with dementia and two were people with younger onset dementia. FG3 involved five participants, all female. Two were care workers, one a care home manager, one a dementia care educator and one a palliative care nurse researcher. All had experience of caring for people with dementia nearing the end of their lives.

All focus groups started with the researchers serving tea and coffee and offering participants food such as fruit and cakes. In this way an informal atmosphere was created in which participants who knew each other could catch up and those who didn’t could be introduced to each other. Formal introductions were made, information sheets reviewed and consent forms signed. Topic guides (Table 1) developed using key themes arising from a literature review [26] were used to structure the focus groups. The focus groups were lively, with participants expressing opinions and experiences quite freely and listening respectfully to each person’s contributions. Each focus group lasted approximately one and a half hours and was audio-recorded with participants’ consent. All voice files from audio recordings were transcribed verbatim. Field notes and notes of debriefing sessions following the focus groups were made to record developments in thinking and researchers’ impressions.

**Table 1:** Focus group discussion points

Group	Discussion guide
People with dementia Recently bereaved family members	1. What aspects of the design of your house/garden are important to you at the moment?
	2. If you have been in hospital or a medical or nursing setting, can you describe aspects of the design of the setting that you liked and/or disliked?
	3. What are the key design differences between a hospital and your home? How do they both make you feel?
	4. What changes would you like to make to a hospital setting that would make you feel more comfortable if you are in hospital or other medical setting?
	5. If you become frail or ill, what are the key things that you would like to have in place to help you feel more comfortable?
	6. Expand on why they would make you feel better
	7. Expand on aspects of design you think would make you feel worse and why?
Practitioners	1. What are the key areas of importance to you when caring for someone with dementia who is frail and/or nearing the end of life?
	2. When someone with dementia is dying, what do you do to care for them?
	3. What are your concerns when caring for someone with dementia who is dying?
	4. From your experience of caring for someone with dementia who is dying, what aspects of the design of the physical environment help or hinder the care you give?
	5. What aspects of the physical environment would you like to improve when you are caring for someone with dementia who is frail and/or nearing the end of life?
	6. If someone was restless in bed, what would you do to help make them more comfortable? How could the design of the setting help or hinder you with this?

### Survey to identify desirable characteristics

Delphi surveys are an iterative method used to reach consensus of a group of experts on a particular issue [32] and have been used to assist in defining palliation in dementia care [7]. In order to reach a consensus on the principles of environmental design that might accommodate the needs of people with dementia nearing the end of their lives, a total of 22 professionals in the areas of architecture and interior design for aged care and end-of-life and palliative care, sourced from a convenience sample of the contacts of the investigators, were invited to participate in a Delphi process. Twenty consented and one who declined offered an alternate expert who accepted.

The 21 experts surveyed included 10 experts in environmental design of care facilities for older people, eight experts in palliative and/or end of life care, one expert in advanced dementia care, one expert in hospital intercommunication and one expert in end of life environments. Experts were from research (7) and practice (12) or both (2). Fifteen were based in Australia and six in the UK. The average number of years of experience providing, developing or researching services for people with dementia was 18.3 (ranging from 5 to 30 years) and the average number of years providing, developing or researching services for people with dementia in the final stages was 13.7 years (ranging from 0 to 30 years).

The list of 11 desirable features for the environment which were identified in the focus groups was developed into a series of questions, each asking the professional to rate their perception of the level of importance of that feature to people with dementia nearing the end of life on a 5-point Likert scale. The questionnaire then asked for examples of how this feature might be seen in practice and for any features that may be missing from the list presented. The questionnaire also asked a number of demographic questions and questions about level of experience and area of practice. The survey was administered using the online survey program, SurveyMonkey. The survey ran for five and a half weeks and one personalised reminder was sent during the survey period. Individual survey completions were not able to be determined as the survey was anonymous. At the closing date, 21 participants had contributed to the survey and 18 had completed all questions.

The list of desirable environmental features was refined following analysis of responses to the first survey. It included a list of the nine features with at least 50% strong support and a second list of six features that were derived from panel member comments. The refined list was then circulated to all survey participants who were asked to consider if any of the features should not be included, to provide any comments if they wished and to provide any additional features if they felt any of high importance were missing. Provision of comments was optional and participants were given three weeks to do this. A total of nine participants contributed to the second round of the survey, eight participants completed all questions.

## Analysis

### Focus groups

Focus group transcripts were read thoroughly alongside audio files to ensure accuracy of transcription and to gain a feel for what participants were saying. Transcripts were then read carefully several times by one researcher to identify initial codes and themes based on the topic guides and issues arising during the focus groups [33]. They were then reviewed by the other researcher alongside initial codes and themes to ensure accuracy. Discussions were held on emerging ideas and themes that would inform the development or adaptation of existing design principles. All focus group data were managed using qualitative data management software NVivo8. From analysis of the focus groups, a list of 11 environmental features was identified to support the care of people with dementia nearing the end of life.

### Modified delphi survey

Expert panel ratings of the importance of each environmental feature were used to refine the initial 11-item list. Features that did not have at least 50% strong support (i.e. 50% or more of participants thought they were very, or extremely, important), where separated out into a second list. Free response examples provided by each participant were organised into items within each main environmental feature. These items exemplified how each feature could be realised in practice. Comments made throughout the survey as well as in response to the question about whether panel members felt any features were missing, were thematically analysed and used to develop a list of additional exemplars and features for the second round of the survey. No additional features were provided in the second round, but comments made by panel members who participated in that round of the survey were incorporated into the exemplars of desirable environmental features to produce the final list.

## Results

### Views of people with dementia and family carers

#### Comfort through engagement

Family carers spoke spontaneously and movingly about how they worked to maintain engagement with the person with dementia they were caring for, up until the end of life. This ranged from engagement with the senses, spiritual engagement and social engagement, with the goal of providing comfort to the person. The two participants with dementia were also clear that being able (and helped) to engage by whatever means was possible would also be important to them as they neared the end of their lives. Participants talked in different ways about how the environment might sooth or comfort the person with dementia. They were clear that attempts must be made to ensure that care was aimed at ensuring comfort through engagement with the senses, even towards the end of life:

*Perhaps if they’re not well anymore, right at the end, and comfort is something that can be adjusted, adjustable bed with, you know, ventilation and a window nearby and, you know, music nearby, so that if you sense that’s what’s of comfort to them, would be important.* (FG1F4)

This family carer describes how she worked to help her husband engage his senses with the things that had been important to him: bird song, sunshine and the scent of flowers:

*We got (husband) out every day that the sun was shining and when it wasn’t freezing cold, he came home mid-winter and we’d put his beanie on and get him into the recliner wheelchair… and out into the grass in the garden… lots of garden, lots of birds. He’s an ornithologist, loved birds, could tell them all by their sound, you know, and we’ve got lots of jasmine, and stuff like that, around so, very early spring, like now, the smell of the jasmine was around and you could see him responding to it. So, in terms of those things, and just feeling the sun, just feeling the warmth of the sun was incredibly important.* (FG1F4)

Using outside spaces to facilitate engagement with the senses was seen as very important by carers, even for those who were nearing the end of their lives:

*I do think it’s… most people like outdoors, there are very few people who don’t like looking at the leaves fluttering on the trees and being outdoors, I think, there isn’t enough attention and enough space for people to be taken outside, to be able to be taken outside, I think, that’s crucial.* (FG1F5)

Spiritual engagement was seen in broader terms by a few participants, for example, this participant with dementia viewed spirituality (in its broadest sense) as a way of nurturing her core self:

*It depends how you define spirituality, I always say you've got your cognition and then you've got your emotions and then there's the inner you. That could be the herb garden or the music or the pets, or it could be your faith system or all of the above. But I think it does becomes much more important* (near the end of life)*, because if you can’t do all of that remembering and factual things, and you can’t do the talking and the emotional, who everybody is, then you really are your true self, and you can be nurtured as your true self.* (FG2F1)

The importance of social engagement was raised by several participants and included engaging with family, friends, the community of residents if in long-term care and pets or dolls. For example, this carer was clear that people, regardless of mobility or frailty, should experience the company of others:

*But, even, in residential care, I don’t think that the focus should be keeping a person in their room, even if they’re no longer mobile, they have to get out of that room, I think that’s terribly important .* (FG1F5)

Others described the sense of peace that can arise with the quiet, gentle, loving company of family:

*But, it was having (his son) sitting next to him, holding his hand, talking about some of the things that were there, just, periodically, feeding him, talking some more, just sitting quietly together, just that sense, gentle light in the room, you know, moderate sounds, just, they were the things in terms of the make up of the environment.* (FG1F4)

In an example of recognising the potential of social engagement for providing comfort, this carer described the comfort her father gained from having the cat sleep on the bed with him:

*Something I did with my dad is he used to get up and wander every night and purely by accident one night the cat got stuck in his room, and the next morning, he actually didn’t get up that morning, and the next morning when I went into his room the cat was curled up in bed with him. And so from then on for the last two years, every night, I put the cat in bed with him, and he actually died at home with the cat around him.* (FG2F6)

As this might not always be feasible, an alternative was suggested by some carers: pretend dogs and cats, which look very realistic and may also meet comfort needs. For example:

*I don’t know, I was just going to say there was a lady here a few weeks back and her family said she was very restless so as soon as they gave her this little (pretend) cat and a basket or a rug or whatever it was, anyway, she just sat there like this and she was…* (FG2F8)

*And she began to open up and speak.* (FG2M2)

Another participant also recognised the importance of being able to engage socially with who or whatever could provide comfort at that particular time:

*I find that where (wife) is, everybody’s different and they have different things. One lady has a doll, a big doll, it’s almost life-like. I thought it was a doll. Others have dogs and octopuses, all kinds of things. But a lady has a little poodle, and she takes it round to the various ones that she knows loves a dog and puts it in the bed with her. And just to see the reaction on people is just wonderful.* (FG2M3)

All of these accounts are founded on the conviction that it is possible to provide comfort to people with dementia until they die and that this can be achieved through engaging the person either through whatever senses are possible or appropriate, through spiritual means and through the company of family, friends or other means such as dolls or pets.

#### Feeling at home/familiar

Feeling at home or the sense that the environment or aspects of it were familiar to the person with dementia was seen as important to participants in focus groups 1 and 2. For example:

*I tend to think that people with dementia do want familiar; it’s the change that is difficult to cope with and the familiar things are personal things, if we’re talking about residential care, to bring in personal things of theirs, whether it was his music, I know my husband did a lot of photography as a hobby… and he had the photographs there…and when he did go into respite, we took the same pictures, I think, that was important to him.* (FG1F5)

This participant with dementia was clear in her wishes for the end of life and referred to the concept of aging in place, where people will live and die in the same familiar place:

*Because the last thing I want to happen to me is to be moved. I want to feel at home.* (FG2F1)

This carer describes the facility where his wife is and stresses the importance of the familiar feel her own possessions give to it:

*Where my wife is at present, she has the most wonderful room,, and they told me when I went there to make it like it was her home. So I brought in some of her paintings and photographs, everything that’s all around the wall, TV. And outside she’s got a door that opens out into a little porch which has a table on it and two chairs, and she can look straight out into the car park and see me coming in.* (FG2M3)

The overwhelming opinion of carers and people with dementia is that care settings must have a homely feel; this will be achieved through having the person’s own belongings, ornaments, pictures, television *etc.* in it.

#### Calm environment

The importance of ensuring a calm environment was stressed by carers and people with dementia, whether this was at home or in a care setting. This participant with dementia was clear she did not want to be in a noisy environment, what was important for her was calm, peace and quiet:

*I think the calm and peaceful environment is…for me, walking into an environment where there’s lots of noise and other surroundings going on seems to affect my coping skills and how I would interact. And I feel that certainly that will stay until the end, the quiet peaceful, serene surroundings seems to be the most important thing, it does impact greatly I feel.* (FG2F9)

This participant with dementia also expressed her distaste of noise and overwhelming stimuli:

*Nobody seems to understand, but it’s visual stuff, visual clutter. When I was visiting last year in a dementia ward, was not only obviously the sound level, the TV and the radio and the staff talking loudly to each other, but it was a smaller area, there were lots of people, lots of tables, people coming in and out and then the occupational therapist had made stuff, which was hanging everywhere and it was just…And then there were loads of those walkers everywhere; it was just visually … really, really stressful. I would just go there for an hour and I’d be exhausted, And I often think no wonder people in nursing homes are just sitting there like that, because I felt like that when I went in, that I wanted just to sit, close my eyes, because it was too much.* (FG2F1)

This was also reiterated by the carers who described how they worked to ensure the environment was calm and peaceful:

*So one of the things that I’d do, we had the candles, not that he could smell anything, but it was that nice soft light, …I’d have the classical music on, which I just left on until he went to sleep at night and, then, I turned it off and it was just all that softness and calm and it’s very hard to be calm when you’re not a calm person but, for eighteen months we managed because you do need that, you need to have no conflict, totally conflict free, totally and utterly*. (FG1F2)

In a similar manner to ensuring comfort through engagement, carers were able to recognise when the person with dementia needed peace, stimulation appropriate to their needs and abilities and an environment free of conflict (excessive noise or visual stimuli).

#### Privacy and dignity

While all participants agreed on the need to ensure the person’s dignity was respected while carrying out care, there were some differences in opinion on the necessity of ensuring privacy – ensuite or shared bathroom, single bedroom or shared bedroom. For carers, privacy also meant private, quiet spaces where they could go to rest or cry. This was an important aspect of the design of a care setting to enable them to keep strong and continue to be psychologically and physically available for the person with dementia:

*Is it possible to have a small space, a private space for carers that…Many a time I have to go and stand in the back corridors between two houses to cry sometimes because I’m so upset, because I can’t do it where* (husband) *is. And here I am standing out in a hallway where the laundry comes in. So in an ideal world could there be, as they have in hospitals, a small intimate space for families, or you can go and you can have a cry and then go back and face it again.* (FG2F8)

From these accounts, respecting dignity and privacy are important and, crucially, become more important as dementia progresses; having ensuite bathrooms ensures that dignity and privacy are maintained when increasing frailty necessitates more personal care.

#### Use of technology

Carers and people with dementia viewed technology as a means of remaining connected to others (family) and of alerting others (practitioners) of a need. For example, one participant with dementia wanted to be able to connect with her husband, and wanted him to be able to check in on her, through a webcam:

*Well, when I get to that stage I would like my husband to be able to check on the webcam.* (FG2F1)

Technology could also be used to engage with the senses, for example a visual projection onto the ceiling for people who are in reclining chairs or confined to bed was suggested as a useful way to ensure a more interesting experience. Other technology, such as sensor mats or monitoring equipment were also suggested as possible ways of ensuring the person remained safe, particularly in busy settings when staff might not have time to regularly check on people.

There was some discussion about safety, particularly with maintaining the person’s safety when they are confined to bed. There was a general feeling that bed rails would be acceptable, if there was a risk the person might fall out of bed, as this participant with dementia said:

*Well, I’d be happy to have that for myself rather than fall. And I was happy to have it for my mum because in the last few days she really needed it.* (FG2F1)

Technology should, however, be used with awareness of how it might be interpreted or understood by people with dementia, for example a hoist might not be well tolerated, as this carer identified:

*… coping with that whole process of losing more, losing more capacities, and so on, when their environment is so confounding for them…and* (husband) *at one point, thought the electronic hoist, in the room, was something that was very fearsome.* (FG1F4)

These accounts highlight the ways in which technology can be used to help people with dementia and their families remain connected with each other and to alert professionals of need, yet they also provide a reminder of the sensitivity with which practitioners and families need to approach the use of technology, so that it is understandable and acceptable to the person regardless of cognitive ability.

### Views of practitioners

#### Practice at end of life

Practitioners spoke in terms of their practice rather than in terms of their understanding of the needs or wishes of people with dementia as they neared the end of their lives. One practice at the end of life seemed to be the setting up of a syringe driver with morphine and other drugs aimed at sedating the person once it was established they were approaching death:

*The drivers really are a great idea*. (FG3F5)

This appeared to be instigated because dying was seen to be an uncomfortable process:

*Well, dying is not necessarily particularly comfortable.* (FG3F1)

But also as a pragmatic response to under-staffing of the setting in which one care worker might be looking after 18 patients on his/her own.

*The workload reduces, you’re not doing PRNs every evening.* (FG3F5)

(PRN (Pro Re Nata) = ‘as needed’. Usually refers to administration of medications.)

Practitioners talked of the consequences of sedating patients as they near the end of life:

*…keep it nice and simple, once they get to palliative with a syringe driver, they usually won’t hit out, they don’t kick, they don’t walk, they won’t bite, they don’t scratch....they are so peaceful and calm and they just lie there, they’re basically asleep the entire time… so it really doesn’t…the environment around them doesn’t matter…* (FG3F1)

These accounts indicate that practitioners’ understandings of the needs and experiences of people with dementia nearing the end of life appear to be influenced by their practice and the impact of their practice on them, rather than on an understanding of their needs and wishes as individuals. If, as is suggested, syringe drivers containing morphine are used, this will inevitably influence practitioners’ views of the experiences of people with dementia as they near the end of life; thus influencing their views on the necessity or otherwise of ensuring the design of the environment meets their engagement, spiritual and social needs.

#### Design to improve working lives

Practitioners had strong views on the extent to which the design of their work settings was safe for their patients and the extent to which it helped or hindered their work.

*I mean I will say, the layout of our dementia floor is ridiculous. Absolutely ridiculous. It just needs to be erased or rebuild a new one. It’s just…as a care staff, it’s a nightmare. An absolute nightmare.* (FG3F1)

Buildings with long corridors and ‘nooks and crannies’ were deemed unsafe as these encouraged patients ‘wandering’. An ideal design put forward by one participant would be a circle so that people would not arrive at a dead end. Some recounted creative ways to disguise dead ends, such as a mural or other feature to hide an area that was off limits to the person with dementia. Wide corridors and wide doorways were seen as vital for easy access for those with wheelchairs, and electronic beds that would raise and lower were also reported as useful.

Practitioners’ ideas for good design were focused on monitoring patients, particularly as they become frailer, and included Nightingale wards (large long wards with beds along each wall) and ‘palliative suites’ which are rooms that are set up to care for patients reaching the end of life. These were seen as appropriate for delivering good care, although this was viewed more in terms of practicalities rather than in terms of how this might influence the experience of people with dementia and their families. For example:

*Palliative care suites are beautiful, I don’t know if you’ve had anything to do with them? They’re absolutely delightful. And more often than not, they have more than one room; there’s like a bedroom and an associated room, so you have space for both the family and the person.* (FG3F3)

Some practitioners also spoke of the preparations they would make when they know the person was dying – they would take out the ‘dying box’ which contained candles, incense, a vase and objects to create a ‘calming atmosphere’.

From these accounts, the design of the building has relevance for practitioners if it can make their working lives easier – if it can allow for easier monitoring of patients, prevent them ‘wandering’ and ensure their safety. While there was some thought into creating a nice, calm atmosphere for the person at the end of life, this was not central to their views about the design of a care setting, possibly because their practice experience is one of caring for people who are sedated and therefore unable to engage with their environment.

#### Systems and institutional influences

A key area of concern for practitioners was lack of staff to provide sufficient care to their patients. There seemed to be a sense of juggling their time between those who were mobile and who needed monitoring and those who were becoming frail and needed more one-to-one care. They recognised the need to have more intensive one-to-one care when someone is dying, but current staffing levels prevent this and this seemed to be a source of frustration for some practitioners.

*You don’t have the resources to… And if you could take two off to look after the one that was dying…* (FG3F2)

Another key area of concern was the funding of aged care and the difficulty of securing enough resources (in a timely manner) to cope with patients’ changing needs. This seemed to require knowledge of the system and strategies to ‘play’ it. Practitioners described a constant battle to secure resources and if they didn’t manage to secure them they would have to do without extra resources (usually more staff) and this inevitably has an impact on the quality of care.

Practitioners’ use of language revealed embeddedness within institutional systems and processes and this was particularly evident when participants referred to patients. The use of words such as ‘dementias’, ‘dementia cases’, ‘the respites’, ‘behaviours’, ‘wheelchairs’, ‘lifters’ indicated they viewed their patients predominately in terms of the tasks required by them, their needs or their disability resulting from dementia rather than as unique individuals. For example:

*I was just thinking of two dementia cases that we have; one who, like you were saying, wandering, wandering, still running around.* (FG3F5)

Practitioners’ accounts of the constraints of the systems they had to work in illustrate the difficulties of thinking beyond the day to day practicalities of carrying out care. This might explain the difficulty they had thinking about design in relation to the experiences of people with dementia who are nearing the end of life.

Notwithstanding the difference in emphasis between the focus group participants, several main themes emerged concerning the provision of:

- An environment that supports the continued use of the senses
- Opportunities for social engagement
- Opportunities for spiritual engagement
- Familiarity and homeliness
- Calmness
- The means to control levels of stimulation
- Opportunities for the family to be with the person with dementia
- Privacy
- The maintenance of dignity by, for example, providing all of the facilities required for personal care
- Opportunities for monitoring of residents by care staff
- Technology, particularly communication technology

### Views of experts in design, end of life and palliative care for people with dementia

Having been presented with the 11 items derived from focus group analysis, the following nine features of the physical environment received strong support (more than 50% of panel members thought they were extremely or very important) in terms of their importance to people with dementia nearing the end of life:

- Support of the continued use of the senses
- Provision of opportunities for engagement with spiritual aspects of life
- Provision of opportunities for social engagement
- Promotion of a sense of familiarity and homeliness
- Promotion of calmness
- Provision of opportunities to be with family
- Provision of privacy
- Fostering of dignity
- Enabling of visual monitoring by staff – via human contact and not through the resident being placed in a public area

Underpinning all of these was the importance for the design of the physical environment to support a personalised approach and a sense of homeliness/domesticity.

The following features were identified by panel members as missing from the original list.

- Provision of access to the outdoors/natural environment
- Access to nature (e.g. plants, natural light, fresh air)
- Support of safety and security- this domain requires further consideration and definition
- A focus on legibility (e.g. ability of staff, residents and visitors to find their way around/know where things are)
- Reduce physical stress (e.g. provision of appropriate beds/mattresses)
- Facilitate nursing care (e.g. facilitate bathing, feeding, going to the toilet, moving and handling.)

Survey participants were invited to comment on the revised list of nine strongly supported characteristics and six additional characteristics. There was unanimous agreement on the inclusion of both the strongly supported and the additional characteristics from the eight panel members who responded to this question.

## Discussion

It was clear from the analysis that participants with dementia and family carers differed from practitioners in terms of what might be important design principles in the care of people with dementia nearing the end of life and in ensuring an optimum experience for the person nearing the end of life. People with dementia and family carers placed a strong emphasis on provision of comfort through engaging with the senses, through remaining socially connected (whether through family, friends, pets or soft toys) and through spiritual engagement. These ideas assume some degree of awareness or ability to engage and family carers worked hard to ensure they engaged with the person appropriately and in a way that comforted them. Practitioners seemed to have a different way of offering comfort – to administer morphine through a syringe driver and this had the effect of sedating the person so that they were no longer aware of their surroundings. The implication of this is that there is no need to work to engage spiritually, socially or through the senses and therefore no need to consider how aspects of design might improve their experience. This seems to indicate a complete, and in the authors’ opinion unjustified, disjunction between best practice evidence based care for people at an earlier stage of dementia, which highlights the beneficial effects of a range of non-pharmacological interventions including person centred care, communication skills training [34], music therapy [35] and sensory (vision, hearing, smell, touch and taste) intervention [36-38], and care when nearing the end of life.

Participants with dementia and carers were clear they wanted to be, and remain in, a familiar environment, with their own belongings and familiar things. Practitioners, however, liked the idea of a dying room to which they could move a person who is dying and in which they could create an atmosphere of calm. Practitioners also liked the idea of a dying box from which they could select items to create a calming, homely space. There is support for the beneficial effects of providing a calm environment in the literature on the Namaste approach to the care of people with advanced dementia. Involvement in Namaste Care has been shown to improve interest in the environment and for residents who are withdrawn or have reduced social interaction; participating in the program decreased some indicators of delirium, and decreased the need for administration of anti-anxiety medications [39]. While this research focussed on people with advanced dementia they were not at the stage where transfer to a dying room would be considered. However it is not unreasonable to extrapolate the benefits of a calm environment, without necessarily a transfer to a dying room, to this group.

Participants with dementia and family carers were clear they wanted care and an environment that ensured dignity and privacy. Most of them were also clear of the need to ensure this increases with increasing cognitive impairment and this particularly related to having easy access to ensuite bathrooms. Practitioners were concerned about the balance between privacy and safety – possibly a reflection of a focus on design to enable fulfilment of duty of care as opposed to facilitating a “good end”. All participants recognised the need for family members to have access to a quiet, private space to rest in and take time out in.

The views of the practitioners are of particular concern as they differ so much from what we would like to think is normal, good practice. However they may be a stark reminder of reality. They recognised their practice was constrained by organisational and institutional factors, such as funding arrangements for delivering care, resource and staffing levels and inadequate design of care settings. They also appeared to be unconsciously constrained by unquestioned practices, such as instituting a syringe driver on recognition that someone is dying and the practice of labelling patients according to their needs or levels of impairment. These seemed to influence their perceptions on the importance or otherwise of aspects of the environment for people with dementia nearing the end of life. A study of 61 care managers’ perceptions and practices toward end of life care in a sample of UK care homes found a range of interpretations of “end of life”, with a focus on the actual event of death [40]. A recent review of the literature found multiple studies observing higher usage of restraint, sedation and tube feeding among people the final stages of dementia than in other terminal illnesses or those who were dying without dementia [26]. This research and the responses provided in this study, suggest that there is little awareness of the benefits of a relationship-based care model with its focus on leadership, teamwork, professional nursing practice, patient care delivery system, resource driven practice and outcome measurement with the patient and family at the centre of all activities [41]. There is a need for increased research, education and approaches to end of life care for those providing care to people living in residential care, especially those with dementia nearing the end of their lives [42].

When presented with the list of desirable features derived from the literature review and expanded on by the focus groups, the panel of experts showed a high degree of agreement on a set of desirable features and were able to identify, and agree on, a set of additional features. Thus, the environment should:

1. Support the continued use of the senses
2. Provide access to the outdoors/natural environment
3. Provide access to nature indoors (e.g. plants, natural light, fresh air)
4. Provide opportunities for engagement with spiritual aspects of life
5. Provide opportunities for social engagement
6. Promote a sense of familiarity and homeliness
7. Provide opportunities to be with family
8. Promote calmness
9. Provide privacy
10. Foster dignity
11. Support safety and security
12. Support staff, residents and visitors to find their way around/know where things are
13. Enable visual monitoring by staff – via human contact and not through the person being placed in a public area
14. Reduce physical stress (e.g. provision of appropriate beds/mattresses, managing odours and temperature)
15. Facilitate nursing care (e.g. facilitate bathing, feeding, going to the toilet, moving and handling, assist positioning and reposturing).

**Fig 2:**
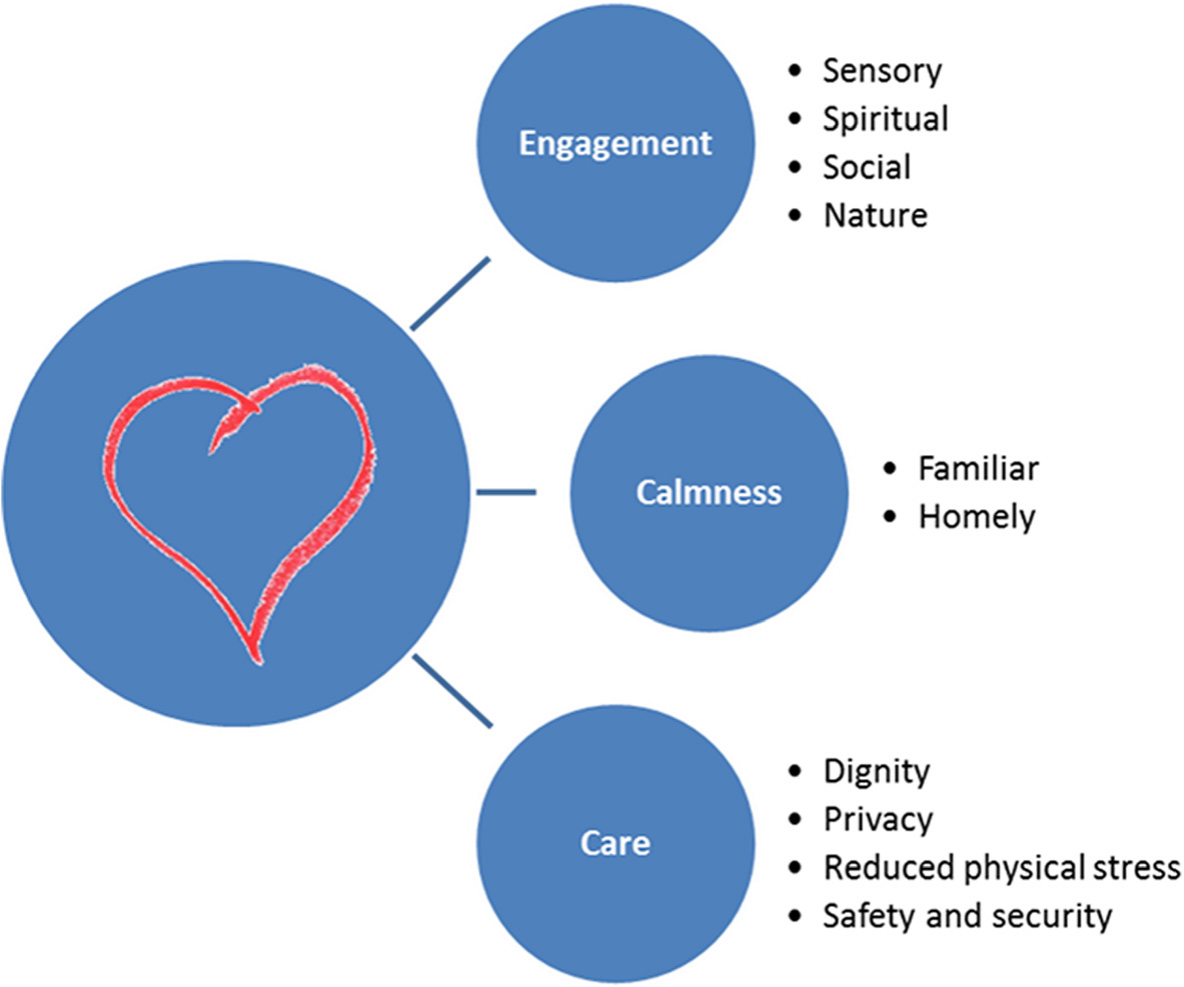
Design requirements for people with dementia nearing the end of their lives

The elements of these design features are summarised in Fig. 2. These can be understood as environmental requirements for meeting the psychosocial needs of people with dementia while they receive care at the end of their lives, and support findings by Godwin and Water who elicited the wishes of 12 people with advanced dementia on what constitutes a helpful environment for end of life care [43]. In consideration of the palliative care needs of those with severe dementia, Hughes [44] compares the psychological needs described by Kitwood [8] with the elements of palliative care described by the World Health Organisation [45] and Addington-Hall [46]. Table 2 extends Hughes’ comparison to include the desirable environmental features. The ease with which the environmental features can be placed within this framework supports the view that they have good face validity.

**Table 2:** Psychological needs, palliative care and environmental requirements

Psychological Needs [8]	WHO definition of palliative care [45]	Aspects of the Addington-Hall approach to palliative care [46]	Environmental needs identified from this study
Attachment	Support to person and family	Importance of sensitive communication	Promote of a sense of familiarity and homeliness
Comfort	Symptom control	Quality of life	Support of the continued use of the senses
			Provide access to the outdoors/natural environment
			Provide access to nature indoors (e.g. plants, natural light, fresh air)
			Promote calmness
			Support safety and security
			Enable visual monitoring by staff – via human contact
			Reduce physical stress
			Facilitate nursing care
Identity	Integration of psychological, social and spiritual	Whole person approach	Provide opportunities for engagement with spiritual aspects of life
			Provide privacy
			Foster dignity
Occupation	Affirmation of life	Respect for autonomy	Provide opportunities for social engagement
Inclusion	Support to person and family	Care of the person and family	Provide opportunities to be with family
			Support staff, residents and visitors to find their way around

(Adapted from Hughes [44], Tables 1 and 2)

### Limitations

It is recognised that the views expressed in the focus groups may not be generalizable to the wider population of people with dementia, their family or professional carers as the sample is small and no steps were taken to attempt to make it representative. The views of people with dementia are represented by the views of two very articulate people with dementia. We have no way of knowing that these views accurately reflect those of people with dementia nearing the end of their lives. They are simply the closest approximation that we were able to access.

## Conclusions

This study identified a set of environmental features that are desirable in buildings used to provide care for people with dementia who are nearing the end of their lives. These features are compatible with the principles currently used in the design of environments for mobile people with dementia and fit well with the psychosocial needs of people with dementia and current approaches to palliative and end of life care. They are offered as a supplement to these principles to ensure the needs and wishes of people with dementia who are less mobile and who are dying are included in future design considerations. This will go some way towards provision of equitable services advocated in policy [4]. We suggest that considering these characteristics as part of a continuum of care will support good practice and offer those with dementia and their families a more positive experience in the last days of their lives together.
